# A 3D Informational Database for Automatic Archiving of Archaeological Pottery Finds

**DOI:** 10.3390/s21030978

**Published:** 2021-02-02

**Authors:** Luca Di Angelo, Paolo Di Stefano, Emanuele Guardiani, Anna Eva Morabito

**Affiliations:** 1 Department of Industrial and Information Engineering, and of Economics, University of L’Aquila, Piazzale E. Pontieri 1, 67100 L’Aquila, Italy; luca.diangelo@univaq.it (L.D.A.); paolo.distefano@univaq.it (P.D.S.); emanuele.guardiani@graduate.univaq.it (E.G.); 2 Department of Engineering for Innovation, University of Salento, via per Monteroni, 73100 Lecce, Italy

**Keywords:** computer methods in archaeology, 3D archaeology, measurement precision in archaeology, similarity metric, information search and retrieval

## Abstract

From archaeological excavations, huge quantities of material are recovered, usually in the form of fragments. Their correct interpretation and classification are laborious and time-consuming and requires measurement, analysis and comparison of several items. Basing these activities on quantitative methods that process 3D digital data from experimental measurements allows optimizing the entire restoration process, making it faster, more accurate and cheaper. The 3D point clouds, captured by the scanning process, are raw data that must be properly processed to be used in automatic systems for the analysis of archeological finds. This paper focuses on the integration of a shape feature recognizer, able to support the semantic decomposition of the ancient artifact into archaeological features, with a structured database, able to query the large amount of information extracted. Through the automatic measurement of the dimensional attributes of the various features, it is possible to facilitate the comparative analyses between archaeological artifacts and the inferences of the archaeologist and to reduce the routine work. Here, a dedicated database has been proposed, able to store the information extracted from huge quantities of archaeological material using a specific shape feature recognizer. This information is useful for making comparisons but also to improve the archaeological knowledge. The database has been implemented and used for the identification of pottery fragments and the reconstruction of archaeological vessels. Reconstruction, in particular, often requires the solution of complex problems, especially when it involves types of potsherds that cannot be treated with traditional methods.

## 1. Introduction

The dimensional evaluation of an object measured by a 3D scanner is a non-trivial task that requires special processing of the point cloud, which is just a raw reproduction of the real object. Direct measurements on point clouds are generally limited to evaluating two-point distances or spherical sizes, quantified as the diameter of the maximum sphere inscribed [1]. These measurements, however, are quite far from determining the characteristic dimensional attributes usually considered, which are associated with specific shape features of the object to be measured (e.g., the diameter of a cylinder or the distance between two parallel opposite planes).

The archaeological fragment is a special case of complex object because it is a part broken off from a find, has a complex shape (that is difficult to classify), and is generally widely damaged due to wear phenomena. The dimensional evaluation of the fragment is, therefore, a rather complex operation, which requires the identification of dedicated shape features. For example, the evaluation of the maximum diameter or the thickness of an archaeological fragment needs the preliminary extraction of the external wall (that is, a shape feature) and the identification of its axis of revolution. Through this recognition process, 3D raw data experimentally acquired from the real object can be segmented into a set of surface patches. These are high-level semantic entities to which some meanings, defined in a specific context, can be associated.

Feature recognition can be performed either manually by experts or as a result of proper processing of point cloud and information on surface texture of the object. In both cases, the recognition process, prior to the dimensional measurement of the artifact, is aimed at identifying external surface portions characterized by specific properties related to geometry, texture, color, pattern, decorative motifs, etc. These features are usually of different geometric types (for example, axially symmetric, planar, cylindrical etc.). Different rules, therefore, have to be specifically designed and implemented to recognize automatically the geometric type of a feature. Depending on the type, it is possible to determine different measurable dimensional characteristics (diameter, thickness, etc.) and situation features (axis, center, etc.) thanks to the development of dedicated methodologies [2]. The algorithmic implementation of these rules and methodologies also allows the automation of the entire measurement process, from the feature recognition to the dimensional analysis of the potsherd, with the advantage of obtaining faster and more accurate measurements than those performed manually by experts.

In [3,4], the authors have proposed a method that adequately supports the recognition process of archaeological features and, through the measurement of the related dimensional attributes, facilitates the comparative analyses and the archaeologist’s inferences by reducing his/her work of routine. During the classification of an archaeological fragment, the archaeologist compares the find’s attributes with those of numerous archaeological classes of objects coming from reference databases in order to search for relationships or affinities between them. The comparative analysis of attributes is also necessary to select fragments belonging to the same archaeological typology (the so-called reunion tasks) or to find correspondences between fragments for the purpose of reassembly.

Measurements of the dimensional attributes, together with the related features, have to be stored in a database appropriately organized to manage this data. By querying the database, the various dimensional and shape attributes of the features can be used to make comparative analyses and various types of research on the set of archived finds.

Faced with the need to properly manage these archaeological data, the paper proposes a database structure that can ensure scalability, storage, integrity and a new way of accessing archaeological information. Another characteristic aspect of the database is the ability to share data and information associated with individual features of the object, which may be the result of the work of many experts who have studied the artifact.

There are various purposes of use for this database: from the simple visualization of the digital 3D version of the archaeological find (which usually interests the visitor of a virtual museum), to the analysis, to the documentation, the classification and reconstruction of the archaeological object (which interests the expert).

The proposed database aims at implementing a new way of documenting and sharing archaeological data within the scientific community. If previously the expert used a hand-drawn sketch with photos and text annotations to document the archaeological find, now he/she can work directly on the digital model of the object by extracting the features of interest manually or automatically (with a feature recognizer) and adding the annotations in the 3D environment. The power of 3D visualization and the potential of the feature recognizer implemented makes, therefore, this data sharing “augmented”. The annotations of the qualified expert, in fact, become part of an expandable database, interrogable and able to convey the entire history of the archaeological investigation conducted on the find.

The case study considered here for the implementation of the new database concerns the classification of ancient ceramics. Archaeological pottery is an effective means of dating the archaeological context and is significant as evidence of technology, tradition, distribution methods, consumption patterns and site formation processes [5].

## 2. Archaeological Feature Recognition

In recent years, several computer-based methods have been proposed to support the semantic decomposition of the archaeological find into features of interest. Basing the analysis and classification of the archaeological artifact not on simple visual inspections but on quantitative methods that process 3D digital data from experimental measurements allows optimizing the entire restoration process, making it faster and more accurate.

Over the past two decades, more and more archaeological research groups have integrated 3D scanners for archaeological research and documentation [6,7,8,9,10]. The acquisition of 3D data, in the form of high-density point clouds or polygonal models, has allowed achieving a very high resolution over the capture of small details, thus making it possible to analyze and document archaeological finds in a more precise and efficient way than using traditional drawings or photographs. Thanks to the ability to convey the entire geometric and textural information on archaeological objects, 3D models are now mainly used to archive, visualize, share and disseminate the archaeological material. Through realistic renderings and 3D prints, these models significantly enhance the promotion of archaeological sites and their artifacts. The production of replicas, even in scale, allows the object to be manipulated for evaluating its possible functional destination; otherwise, it simply allows non-experts to get in physical contact with the testimonies of the past.

Finalizing the use of the 3D model to a simple visualization of the archaeological object, however, is highly limiting. The 3D model should be seen, more generally, as a container of information associated with specific semantic entities (or features) in which the artifact could be decomposed when comparative analyses are performed among finds. Several categories of features can potentially be identified in the ancient object, which are contextual and consistent with a conceptual interpretation proper of the archaeological scope. The feature recognition may also respond to the need to order the archaeological material in different ways based, for example, on geometry, surface texture, decorative motifs, etc. Thanks to it, the 3D point cloud, generally used in archaeological practice only for the 3D rendering of the find, is transformed into a virtual object “augmented” by high-level semantic information useful for many purposes, such as:

- the taxonomic and morphological study of the find based on quantitative methods capable of overcoming the deficiencies of the traditional approach, in particular in relation to the need to objectify and extend the observational units’ evaluation and comparison [11];
- the repeatable and accurate measure of certain properties associated with the features present in the find, that are useful for making automatic comparisons and assessments on the uses of the object and to recognize ancient standards;
- the support to the 3D reconstruction of the object from fragments, reducing the research effort of matching fragments;
- the annotation of the object features and the via web sharing of the detailed information and archaeologist notes stuck on the digital model of the artifact.

Translating the traditional approach to the study of archaeological pottery into a method based on automatic feature recognition is a non-trivial process for several reasons. Firstly, complex and non-analytical shapes, usually attributable to axially symmetric geometries of a generic type, characterize the archaeological artifact. The finds, also, even if repeatable, are unique handmade pieces with surfaces usually damaged and worn, so that their geometric properties are often altered or lost.

The automatic recognition process of archaeological features requires the implementation of a feature recognizer, that is, an agent segmenting the surface of the ancient object according to some rules properly designed to identify portions of the surface, which may be associated with a specific archeological significance. The most challenging aspect, in the implementation of the feature recognizer, lies in the need to define a set of features in accordance with the way in which archaeologists perform the conceptual categorization of ancient ceramics. The formulation of rules, which translate expert human knowledge into a series of mathematical algorithms by which to perform automatic feature recognition from 3D models, is a rather complex problem.

### Overview on Automatic Feature Recognition Methods and Databases from Archaeological Pottery

A large group of methods, in the related literature, performs automatic identification of the longitudinal profile from the high-density 3D model of the axially symmetric fragment. Archaeological pottery was commonly made on a potter’s wheel so the longitudinal profile, which is the cross-section of the fragment with a plane going through the rotation axis of the pot to which the sherd belongs, provides a convenient and concise representation of the entire vessel geometry. These methodologies can be further subdivided into three sub-groups:

- segmentation-based methods;
- shape descriptor-based methods;
- direct matching methods.

Segmentation-based methods first identify the longitudinal profile of the fragment and then segment it into 2D geometric features such as rim, wall, and base through a set of rules based on expert human knowledge and profile curvature. A set of characteristic points is extracted from each profile. Through these points, some dimensional ratios, related to specific parts of the sherd profile, are measured and compared to determine whether, for example, the fragments recovered from a given archaeological excavation fit or not to a specific type of ceramics [12]. The shape descriptor-based methods perform pottery characterization using one or more shape descriptors of the 2D profile and possibly the presence of appendages (typically handles and supports) [13,14,15]. These descriptors (such as radius, tangent and curvature), although easy to calculate, do not show a clear correspondence with the elements of knowledge generally used by archaeologists to analyze and classify archaeological finds. In direct matching methods, finally, classification is based on a direct comparison of the longitudinal profile, extracted from the 3D model, with those of a reference database [16,17,18,19,20].

To ensure that datasets extracted through these methods support the scholar during the documentation and classification of the archaeological find, it is important to store them on adequately organized digital archives. The Pottery Informatics Query Database (PIQD) [12] is an example of a digital repository capable of archiving both vector images of 2D profiles from hand-made drawings on published volumes and 3D models of archaeological ceramics experimentally scanned. The database contains various types of mathematical representations used to describe ceramic longitudinal profiles. Three mathematical functions are particularly associated with each profile, mapping radius, tangent and curvature respectively in terms of the arc-length of profile. The analysis of the similarity between profiles is measured in terms of the Euclidean distance between the three above-mentioned representations. In addition, some weights are attached to each representation, which can be adjusted during the various steps of the comparison between potsherds. The differences between these mathematical representations were used in [12] to develop a novel solution for automated construction of ceramic types from a specific geographical region.

The analysis of the similarity between fragments, using the longitudinal profile as the only element of comparison, is effective in the specific case of potsherds characterized by a simple axially symmetric geometry. Archaeological pottery, however, often shows several details, not necessarily axially symmetric, which are significant for historical and archeological investigation and classification purposes. These features result, for example, in traces on the potsherd surface, which can be left by the action of fingers or by tools, either intentionally, such as inscriptions and decorative motifs, or involuntarily, such as working signs. More recently, some methods have been proposed that can improve the visualization of these detail features through the calculation of shape descriptors of the fragment surface [21]. In [22], parabolic contours are used to help in the reconstruction of ceramic artifacts recovered from an archaeological excavation in a fragmented state with missing pieces. The relief edges are also extracted from archaeological artifacts to automatically translate 3D models into 3D line drawings that can imitate manual archaeological draftsmanship. In any case, these are shape descriptors, which, although easily calculable by the geometric-differential properties of discrete 3D models of archaeological fragments, show no correspondence with the shape features considered by archaeologists in the analysis and classification of archaeological finds. The colored and enhanced images are also generated algorithmically in order to emphasize respectively the salient shape attributes of the artifacts and the 3D details that would be otherwise difficult to discern [23]. In [24], several datasets, during the automatic workflow for the generation of various types of graphic illustrations from archaeological pottery, are created and stored in a digital repository. These datasets identify, in addition to the longitudinal profile and the axis, different types of surface features, classifying them into external or internal, axially symmetric or not, surface irregularities or not. Surface irregularities, generally including plastic decorations, fractured surfaces and technological traces, are very useful and interesting elements for the study of the pottery-making tradition and specialization. The authors of [24], however, do not develop any methodology for recognizing these features in an automatic and differentiated manner, merely proposing two methods of display to highlight them in their entirety. The final objective of the study is, in other words, to provide an enhanced view of the sherd, which, however, does not allow selective recognition and quantitative analysis of the extracted features. 

In [3,4], the authors propose a methodology capable of extracting a wide range of quantitative information for several types of archaeological features automatically recognized by the fragment surface, described in the form of a manifold triangular model. Extracting high-level semantic information requires preliminary estimation of geometric-differential properties, such as normal and principal curvatures at each mesh vertex. Another important pre-requisite is axis evaluation. Several methods for axis estimation, which differ in the use of specific geometric properties of axially symmetric surface, have been implemented in [25] in order to identify the most reliable algorithms for the archaeological context. Firstly, the methodology automatically decomposes the surface fragment into axially symmetrical features and not. Various archaeological features, such as rim, wall and base, are then recognized by the axially symmetric portion of the fragment. Each of these features can be further specialized in a set of specific sub-types. The non-symmetrical part of the fragment includes handles, fracture surfaces and chips. The methodology, by investigating the principal curvature (maximum in absolute value) at mesh vertices, is also able to automatically recognize various detail features with an approximately constant radius. In order to overcome the limits of a recognition affected by uncertainties (the estimates of the principal curvatures are influenced by a high variability), the rules for the feature segmentation have been implemented by a Fuzzy approach. Through the dimensional analysis of these detail features, some interesting inferences can be drawn about the technologies used to manufacture them [26].

The feature recognition and the classification of archaeological fragments are at the center of several research initiatives, including two European-funded projects. The project Gravitate [27], completed in November 2018, aimed at creating a workbench of tools and services that, integrating the analysis of geometric and semantic data, help archaeologists in:

- recomposition of fragmented heritage artifacts;
- identification and assembly of fragments/parts that were separated across collections;
- reassociation of cultural heritage artifacts that have common features, allowing one to deduce new knowledge and understanding of past societies.

The project also provides for the activation of a second phase aimed at creating a reference database of 2D drawings of archaeological ceramics already codified (for example, the black-painted ceramics from the Morel 1981 repertoire) and with respect to which to compare the longitudinal profiles extracted from 3D models of scanned potsherds.

The project ArchAIDE [28] is probably the most significant example of a full application, which seeks to redefine digitally the entire canonical pottery classification process. It is a project funded by the European Union Commission under Horizon 2020, the main objective of which is the creation of a new app, available on mobile devices (tablets, smartphones) and desktop computers, for automatic identification of archaeological pottery from a single picture of a sherd. This tool is designed to support classification tasks during on-site work and post-excavation analysis, maintaining the consolidated working methodology of archaeologists. The recognition system is based on a deep learning approach that, starting from a photograph taken by a mobile device (smartphone, tablet, etc.), takes into account two distinct but fundamental elements in the classification of ceramics: shape and decoration.

## 3. The Proposed Database

**Figure 1:**
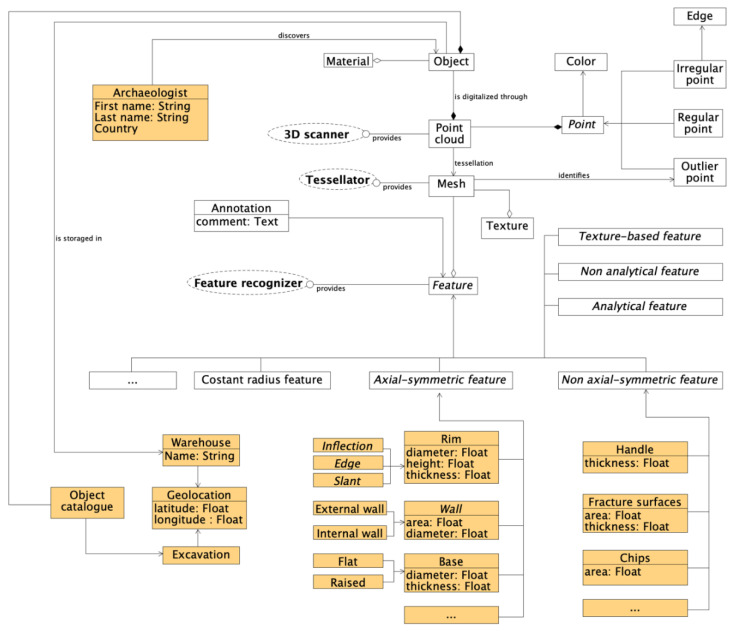
UML diagram of the database.

The database organization is illustrated in Figure 1. Many of its entities have been defined in previous research by the authors [3,4,29,30]. These are geometric features that allow treating many categories of objects, making the proposed application suitable for a large range of uses that require to store and provide geometric data and meta-data. The specific case, considered in this work, refers to the archeology and specifically to the archeological pottery found in fragments.

Two modules make up the database. The first is the main core and is highlighted in Figure 1 by entities shown inside blocks on a white background. Once scanned, the real object is described by a point cloud and its tessellated surface. The surface is segmented into some specific shape features. All the entities present in the database are related to each other using the relationships depicted in Figure 1. A secondary module, called Potsherds and shown by orange blocks, is dedicated to dealing with the specific features typical of this category of archaeological objects. Pottery entities are very specific to the purpose of the application and require an adequate database structure. These entities derive from and are connected to the main core, as they cannot be defined in the absence of the main raw geometric data (point cloud and tessellated surface). The entities in the second module can be removed from the database without compromising the integrity of the primary module.

Three external services, highlighted in the UML notation in Figure 1, are adopted to populate the database with data. The first is a 3D scanner, whose role is to extract a 3D point cloud from a real object. The second is the tessellator that creates the mesh and stores it as ASCII text. Finally, a feature recognizer extracts from the mesh all semantic features, which are significant in the context of interest. The feature recognizer is a key element that regulates the behavior of the application and the knowledge stored in the database.

**Figure 2:**
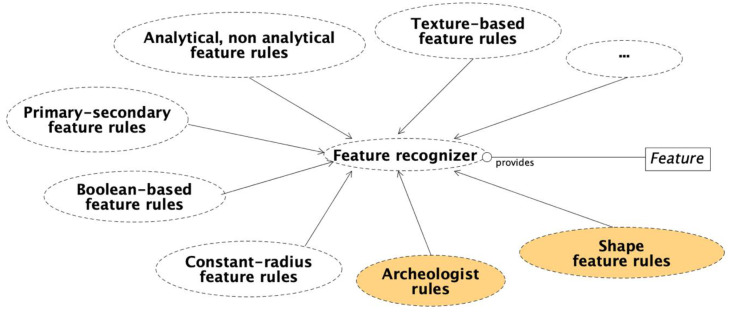
Feature recognizer service.

Despite the concise description of Figure 1, the feature recognizer is a complex external agent, which operates according to a set of user-defined rules, allowing it to recognize specific classes of features. Figure 2 shows in detail the several categories of rules for the feature recognition used in this work. The feature recognizer consists of several original Matlab applications that exchange data with the described database and populate it with the results of the recognition process.

The archaeologist, interacting with the database, can define the category of rules to use for the automatic recognition of specific features; alternatively, he/she can also manually segment the features deemed relevant and annotate them. Indeed, an expert archeologist is undoubtedly the most capable and reliable recognizer of archeological features. This is why the proposed application allows the archaeologist to interact with the virtual version of the find in order to be able to segment the features from himself/herself and annotate them. In addition, the archeologist can observe the find enriched by the information provided by the feature recognition process and stored in the database. He/she can, also, access the annotations provided by other experts, archived in the database, add his/her own, and associate them with the various features. Archaeological features often refer to attributes that cannot be captured by 3D scanning (such as slip opacity, compactness of the paste, temper, etc.) and which, to be evaluated, require the direct interaction between the specialist and the archaeological object. The database is also able to manage these types of information through the insertion of annotations by the expert. Obviously, the data related to the identity of the archaeologist who carried out this type of survey are also included.

**Figure 3:**
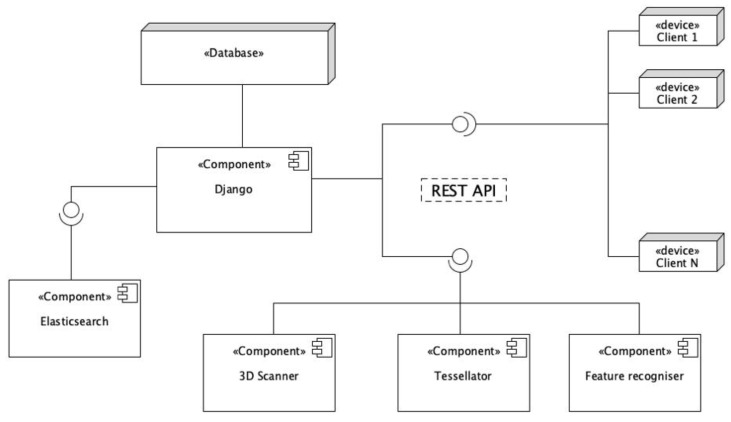
Application component diagram.

Feature recognizers are certainly destined to evolve over time, in line with the development of new data processing methods and the integration of different types of data. Consequently, the database cannot be limited by the current ability of the services to recognize features, but on the contrary, additional feature categories should be implemented with minimal effort. Scalability, flexibility and high-modularity, in other terms, must represent three key database properties described in this paper. For this purpose, the proposed application structure is defined according to the model-view-controller (MVC) pattern. Hence, a powerful and flexible open-source web application, called Django [31], fully accomplishing the MVC logic, has been used to implement the data structure described above. It is a Python-written framework, which allows the rapid and incremental development of web-based applications. One of the main advantages of using it is its smart object relational mapper (ORM) that helps developers to interact with databases, allowing the transfer of data, stored in databases such as MySQL or PostgreSQL, in objects commonly used in the application code. In addition, a large set of external python libraries are available for this framework, included in the Django Rest Framework. This allows implementing a representational state transfer (REST) interface quickly and efficiently, in order to make external web-based applications interact with database objects and business logic. In Figure 3, a high-level view of the configuration of the components is depicted. Devices and external components are able to communicate with the main one, Django, using the REST API. JavaScript object notation (JSON) data-interchange format [32] is adopted for data communications between different components and devices.

To enhance the search capabilities of the application another open-source component, called Elasticsearch, has been interfaced with that of Django. This last is a distributed, open-source search and analytics engine for all types of data, including geospatial, numerical structured, unstructured and textual. Thanks to its implementation in the proposed system, powerful and complex queries, even between not-homogenous data sets, can be performed with a very low effort. Elasticsearch and Django have been interfaced using the Django-elasticsearch-dsl-drf open-source module.

For the case of pottery sherds, the feature recognizer requires an average time of ten minutes for the processing of each object. This time includes the computational cost to recognize the features and enter the data into the database, using the REST API interface. This last operation has been carried out using the web options and web writing objects, already available in Matlab resources. The POST request, executed by the web-writing library to send data to the database, is the most critical operation in terms of time of the entire process.

Even more important than the data structure is how to make these entities accessible and usable by external actors. Most of the information stored in the database is characterized by a high level of abstraction, due to the significant level of specialization required by the external services providing the information itself. Therefore, no existing tools are suitable for this application without some modifications being necessary.

**Figure 4:**
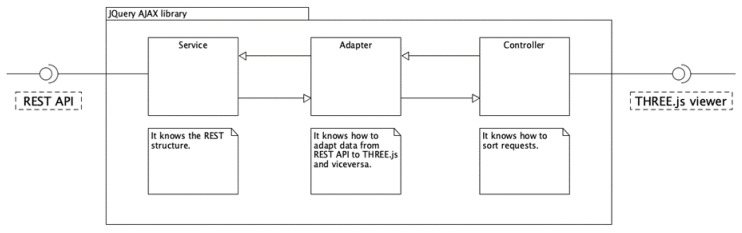
JQuery AJAX library description.

In the application proposed here, THREE.js [33], a powerful WebGL framework, has been adopted to build up the application front-end. Starting from a model viewer, available on GitHub and THREE.js-based [34], the code structure and visual interface have been deeply customized in order to satisfy the desired software specifications. In particular, a new AJAX, JQuery-based, library has been written to interface the THREE.js model viewer with REST API. Figure 4 describes the structure of JQuery AJAX library, fully compliant with general responsibility assignment software patterns (GRASP).

**Figure 5:**
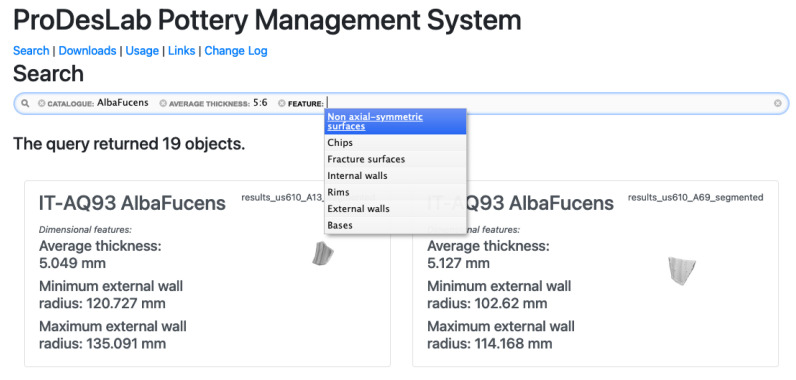
Web interface of search page.

In Figure 5, a snapshot of the custom-developed, browser-based search interface, is depicted. The main search bar is proposed to the user, who can activate several filters associated with the various model entities, even simultaneously, by typing on the keyboard or using the mouse. After the filter selection, a query is sent to backend components through REST API. If some results are returned, the user can click on them to interact through the object viewer module, described in the following.

**Figure 6:**
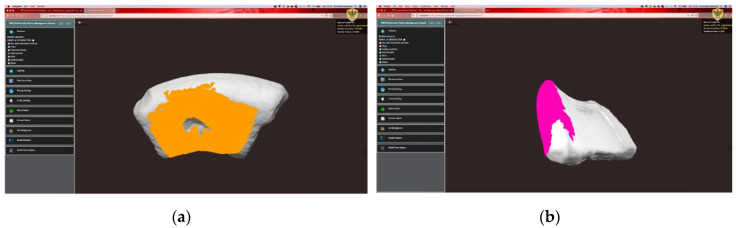
Web interface of object viewer. (**a**) The internal wall has been selected as displayed feature. (**b**) The rim is displayed.

Figure 6 shows the web interface of the object viewer. First, the required model, in the form of an .obj file, is loaded into the browser. When the loading is complete, a preliminary set of information on the object is already available for the user, such as geolocation of warehouse and excavation, or mesh properties. In addition, the object can be navigated in 3D. On the left side of the interface, a set of toolboxes has been made available for the user. These allow making user interacting with the web interface, changing the display options, or the set of features to display.

## 4. Archeological Features on Pottery: Methods and Measures

Each fragment is classified according to the nine different types represented in Table 1 and defined in [3]. These types, respectively named A, B, C etc., differ by the sherd position within the pottery. For each of them, specific ceramic features can be found.

**Figure 7:**
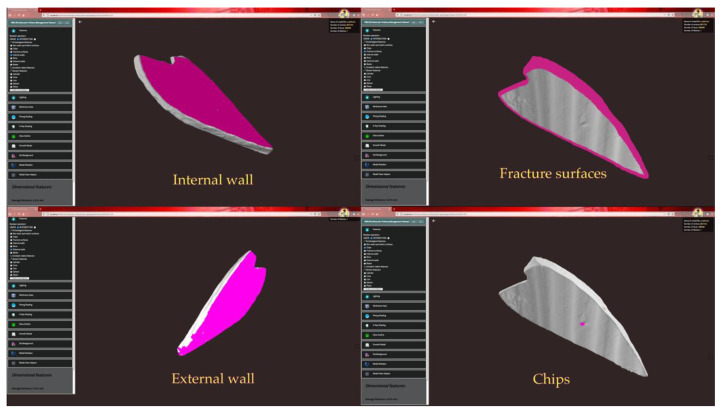
Four screenshots of the application and some recognized features in a type-A sherd.

An example of a type-A fragment is shown in Figure 7, which also displays the user interface developed for the application. Four types of ceramic features, magenta-colored in the figure, are automatically recognized for this object: the fracture surfaces, the external wall, the internal wall and the chips. Being basically a piece of pot wall, the type-A fragment is not generally considered by archaeologists, as it is difficult to identify its axis and profile. Indeed, the lack of ceramic features, such as base and rim, makes this type of fragment challenging to analyze with traditional tools.

Table 2 briefly describes the ceramic features identified by the proposed method. These surface elements of the fragment are significant in the archaeological context and may be useful for further archaeological processes involving the sherd (such as pottery reconstruction, documentation and detailed annotation of potsherd, comparisons and similarity analysis, etc.).

Ancient pottery was commonly made on the potter’s wheel, which gives the archaeological ceramic artifact a substantially axially symmetric shape. External (EW) and internal walls (IW) are typically axially symmetric surfaces. Other axially symmetric features are the rim (R), the neck, the base (B) and some types of foot. On the external wall, however, various non-axially symmetric elements can also be found, such as bas-reliefs or stamped decorations, ribs and graffiti.

The inner wall, which is not always visible, is important to identify the axis and measure certain properties, such as wall thickness and vessel capacity. This feature is generally ignored in ceramic studies unless it presents decorations or working signs of interest for archaeological investigation.

The rim is the top of the ceramic, near the orifice, which connects the external wall to the internal one. There are several types of rim (R) commonly found in archaeological ceramics. To recognize this type of feature, it is assumed that the rim contains the extreme upper part of the artifact and the adjacent rounds. The base B is the lower part of the surface of the ceramic artifact, which extends from the extreme bottom to the axis.

Non-axially symmetric ceramic features include morphological elements deviating from axially symmetric geometry, chips and fracture surfaces. The morphological features (M) are typically parts connected with the external wall of the pottery, such as handles, lip, and ribs. Some bas-reliefs or positive decorations are also included in this type of feature. Chips (CH) and fracture surfaces (FS) are special types of ceramic features that identify damaged parts of the artifact. Fracture surfaces can be useful for the reconstruction of the entire pottery. By querying the database, it would be possible to compare the fragments and identify those with matching fracture surfaces.

**Figure 8:**
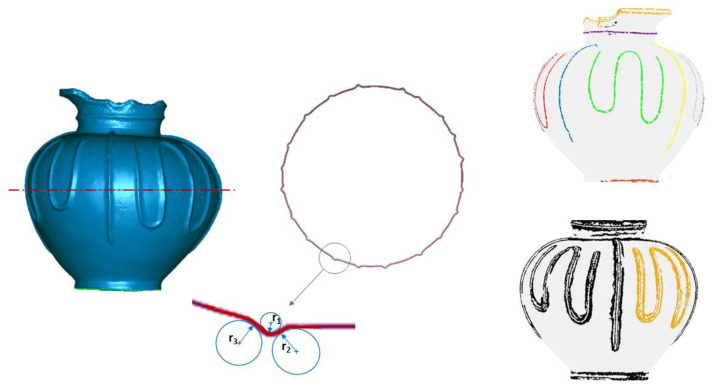
CRS segmentation of an archaeological pot. The different colors are used to highlight the non-adjacent CRS features [4].

The constant radius sweep (CRS) features identify a particular class of ceramic features usually related to engravings, working signs and decorations. These features are not necessarily axially symmetric and are significant for historical and archeological investigation. From the geometric point of view, they are characterized by a constant cross-section, approximated by one or more circular arcs, which develops along a sweep curve. The feature CRS can be made by finger action or by tools both intentionally, as in the case of inscriptions and decorative motifs, or unintentionally, as the signs left by the work. A feature CRS can also derive from the application of sweeping geometries of constant radius section in clay. The sweeping action results in concave or convex traces on the ceramic surface. Examples of concave sweeping features are engravings, graffiti, impressions/stampings, etc. Convex sweeping features are plastic and molded reliefs, etc. In Figure 8, an example of CRS segmentation is depicted. More details on CRF recognition are available in [4].

**Figure 9:**
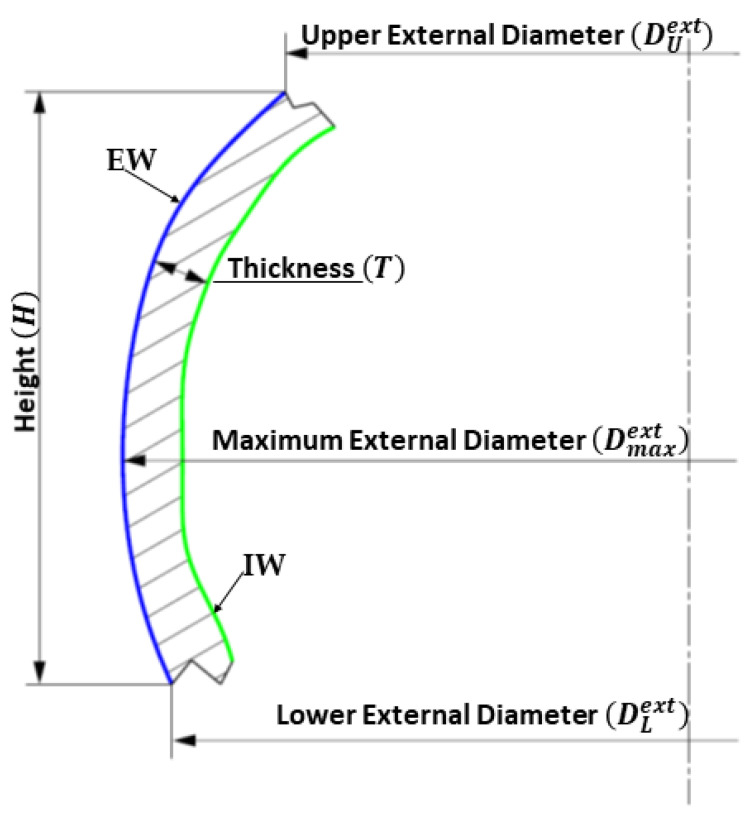
Main geometrical and dimensional features recognized in an archeological sherd.

Specific derived geometric elements can be associated with each feature; they can be point, line, plane and profile. For example, an axially symmetric surface has its axis of symmetry, a straight line, which is a derived feature. Depending on the type of ceramic feature, various specific intrinsic dimensional parameters can be associated. Some of these dimensional parameters are shown in Figure 9. The IW feature extracted from a type-A sherd is dimensionally characterized by the three diameters shown in Figure 9.

All this information is stored directly in the database and, therefore, can be used to query it and perform comparisons between different sherds based on certain specific criteria.

## 5. Experiments, Results and Discussion

**Figure 10:**
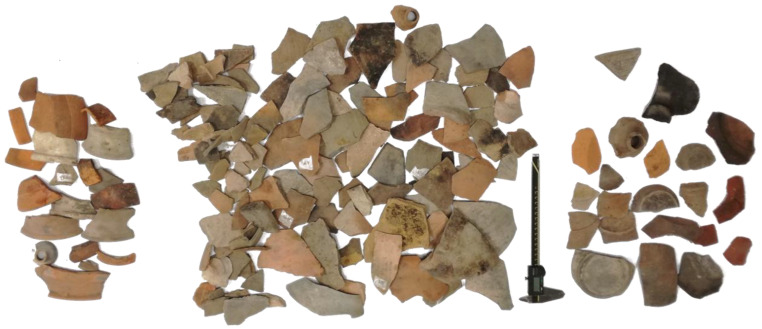
The collection of potsherds from Alba Fucens excavation.

In order to test the capabilities of the application, a case study is considered, relating to Roman ceramics found in fragments at the archaeological site of Alba Fucens (Italy). This consists of a collection $F_{0}$ of ninety-seven potsherds, shown in Figure 10 and experimentally acquired by a 3D scanner FARO (Edge ScanArm) with a laser line probe (accuracy 2σ = 0.0688 mm, sample rate S_r_ = 0.25 mm). The acquisitions have been made by appropriately choosing the position and orientation of the potsherd with respect to the scanner. This is useful for minimizing the number of placements required for the 3D reconstruction of the archaeological find and therefore reducing the errors inevitably associated with the registration process, which combines the different acquisitions together under the same coordinate system. In this experimentation, the registration of the point clouds is performed by the iterative closest point algorithm with a maximum value of 0.05 mm for the mean distance between the two-point clouds. Furthermore, the raw data produced by the 3D scanner cannot be used directly without adequate processing, which typically includes several processes, such as decimating point clouds and filtering. The point clouds decimation aims to obtain a uniform density sample rate of 0.25mm. The data smoothing is performed by a Gaussian filter set to produce a mean displacement of the points not exceeding 0.02 mm.

The mesh generator then processes the point clouds, so that the tessellated models are obtained and sent in input to the feature recognizer. The data resulting from the recognition process shall be stored in the database.

The purpose of the experiment is to identify, from the set of fragments $F_{0}$ in input, the sets of potsherds $P_{k}\subseteq F_{0}$ potentially attributable to the same pottery. To this end, two dimensional characteristics have been chosen to query the database and identify fragments with similar characteristics; they are the thickness and the diameters. In what follows $T^{j}=\left[T_{min}^{j},T_{max}^{j}\right]$ and $D^{j}=\left[D_{min}^{j},D_{max}^{j}\right]$, with $D_{min}^{j}=min\left\{D_{U}^{ext}{}^{j},D_{L}^{ext}{}^{j}\;\right\}$ and $D_{max}^{j}=D_{max}^{ext}{}^{j}$ (the meaning of $D_{U}^{ext}{}^{j},D_{L}^{ext}{}^{j}$ and $D_{max}^{ext}{}^{j}$ has been reported in Figure 9), are respectively the thickness and diameter range for the j-th potsherd.

**Figure 11:**
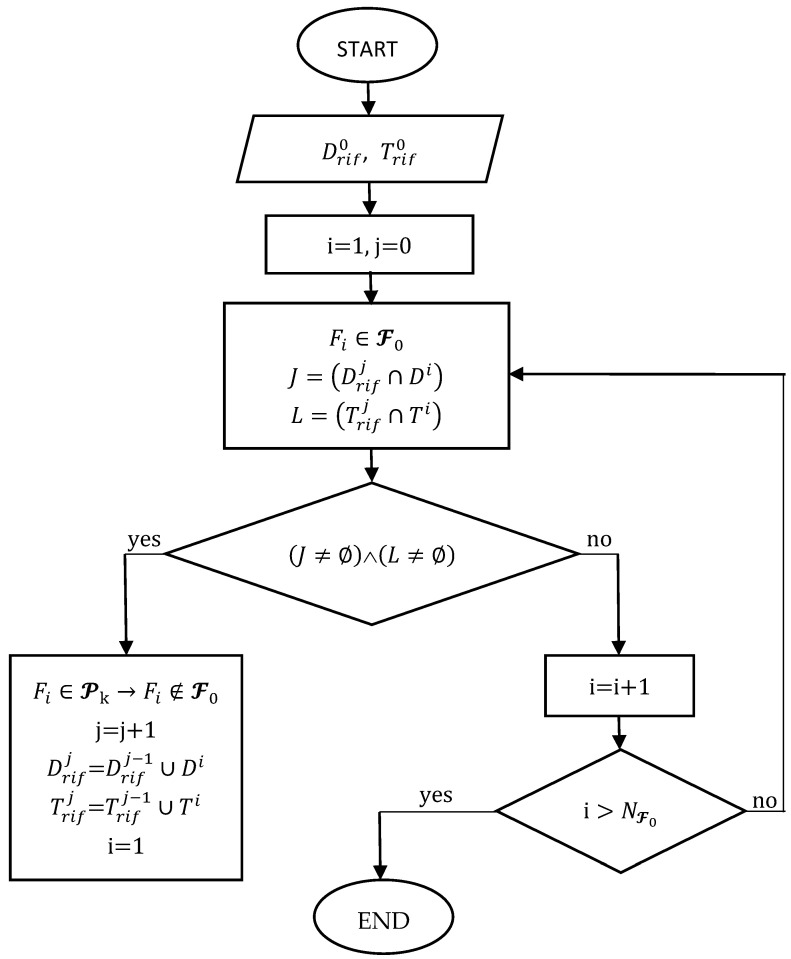
The flowchart of the algorithm.

The flowchart of the algorithm implemented here is shown in Figure 11. A fragment $F^{0}{}^{i}\in F_{0}$, with dimensional intervals $T_{rif}^{0}{}_{i}$ and $D_{rif}^{0}{}_{i}$, is chosen as a reference and then potsherds are identified with dimensional ranges $D^{j}$ and $T^{j}$, which satisfy the relationships $\left(D_{rif}^{j}\cap D^{j}\right)\neq \emptyset$ $T_{rif}^{j}\cap T^{j}\neq \emptyset$,. The progressively identified fragments are removed from the set of fragments $F_{0}$ and grouped into the set $P_{k}$ of fragments potentially attributable to the k-th archaeological vessel. As long as $P_{k}$ contains only the reference fragment, the reference ranges are $T_{rif}^{0}{}_{i}$ and $D_{rif}^{0}{}_{i}$. Whenever the overlap of the two-dimensional ranges is verified for a generic fragment j, the reference ranges $D_{rif}^{j}$ and $T_{rif}^{j}$ are expanded to include the intervals $D^{i}$ and $T^{i}$ of the newly inserted fragment in the set $P_{k}$. The algorithm ends when none of the remaining fragments (in number equal to $N_{F_{0}})$ can be potentially allocated.

**Figure 12:**
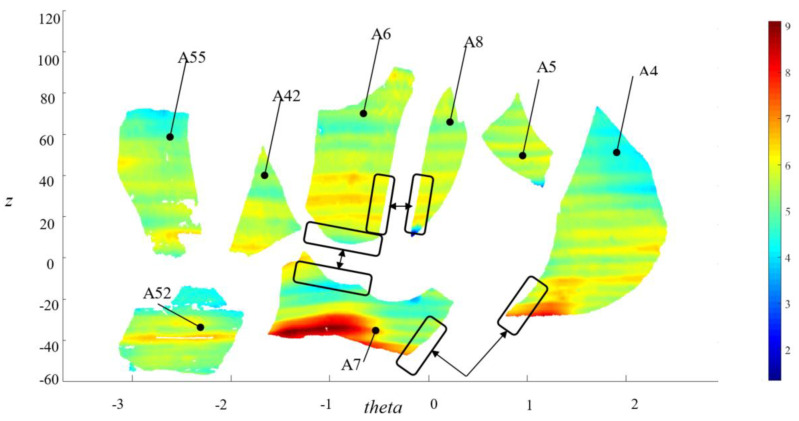
Software interface for pottery reconstruction: matching of the fragments’ profiles.

**Figure 13:**
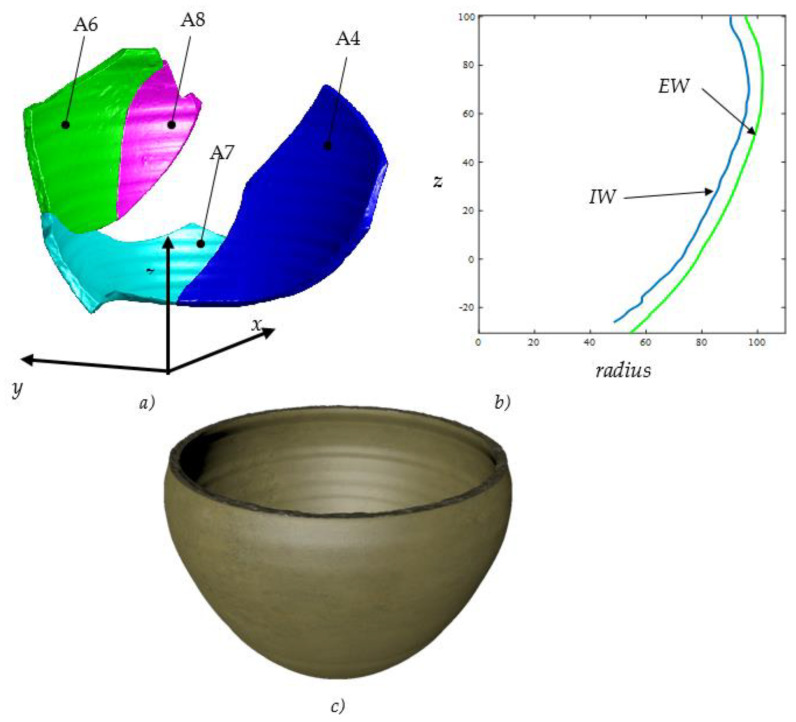
(**a**) The simulated assembly of the four archaeological fragments identified by the algorithm implemented. (**b**) The longitudinal profile and (**c**) the rendering of 3D virtual reconstruction of the archaeological vessel.

By querying the database with thickness and diameter, a set of eight fragments has been identified, potentially belonging to one of the possible potteries $P_{k}$ originally contained in the collection of sherds $F_{0}$ shown in Figure 10. These fragments are all of A-type and are shown in Figure 12. A proprietary software was then used to investigate the matching of the fractured surfaces of these potsherds, discovering, finally, four fragments matched to each other. Te simulated reconstruction of these fragments, the longitudinal profile and the rendered 3D virtual reconstruction of the archaeological vessel are shown in Figure 13a–c.

The result shown in Figure 13 is not so obvious, considering that the four fragments all belong to the wall of the vessel, and that, generally, this type of fragment is not considered by archaeological investigations and ends up accumulating in warehouses. It is well known, indeed, that, through the traditional approach, it is not possible to estimate the axis and therefore to evaluate the profile and the diameters of type-A fragments.

## 6. Conclusions

Nowadays, the study and classification of large quantities of ancient pottery are performed with the traditional human-based method also to perform routine activities. The human ability to assess quantitative elements is scarcely repeatable and depends largely on the experience and capabilities of the archaeologist. In some cases, the traditional approach cannot be used to assess certain types of ceramic fragments, such as those that have no rim or base. As a result, information on shape and morphology may be uncertain and incomplete, because only a part of the fragments found in a site is fully studied.

This paper proposes and describes a new approach to the digital archiving of archaeological finds, able to store and manage the wide range of quantitative information automatically extractable from discrete models of archaeological fragments. This approach combines different technologies (3D scanning, automatic feature recognition, identification and extraction of significant dimensional characteristics) with a database appropriately designed to be expandable and integrable as soon as new archaeological features and/or more performing recognition and measuring tools will be found. Another important characteristic of the database is that it is usable on the web. On the other hand, the long times required for the 3D acquisition of the archaeological material and the processing of the measured data are currently the main limitation to the use of this approach. The acquisitions have to be accurate and have to minimize the errors related to the registration process of the point clouds.

The main functionalities of this 3D information system have been tested in the storage of the data obtained by applying this new approach to the ninety-seven Roman archaeological potsherds, found at the site of Alba Fucens. For this type of finds, it is possible to recognize automatically a large set of archaeological features by virtue of the property of axial symmetry that characterizes them. The proposed database, however, is suitable to record 3D information also from generic archaeological finds.

Although this database was structured to consider different types of information, the experimentation, described in the article, concerned exclusively the geometry of the archaeological find. In the future, the extraction of other information will also be investigated, in particular those relating to the surface appearance of ancient artefacts (such as texture, color, etc.).

The final objective of the work is to facilitate the documentation and investigation of archaeological finds by providing new tools that can improve the analytical skills and stimulate the inferences of the expert. The identification and reconstruction of the ceramics, which are the objectives of the case study considered here, are only two of the problems that can be addressed using the information stored in the proposed database. Proper management of this information is also useful for the creation of virtual museums and for the sharing of archaeological knowledge, as well as of hypotheses and ideas for the study of archaeological finds. The database can be queried to group fragments recognized as similar, based on size and/or morphological similarity. Further queries, duly formulated, can also be implemented for the classification of ceramics according to specific properties.

## Figures and Tables

**Table 1:** Sherd types.

Type	A	B	C	D	E	F	G	H	I
*Example*	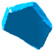	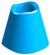	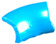	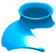	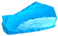	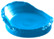	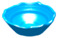	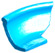	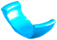
*Position*	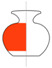	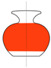	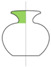	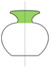	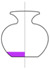	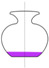	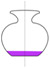	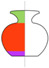	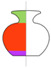
*Features*	IW, EW, FS	IW, EW, FS	IW, EW, R, FS	IW, EW, R, FS	IW, EW, B, FS	IW, EW, B, FS	IW, EW, B, FS	IW, EW, R, B, FS	IW, EW, R, B, FS

**Table 2:** Categories of ceramic features deriving from the sherd surface segmentation.

Ceramic Feature		Detail Classification	Derived Geometric Entities	Dimensional Parameters
**Axially Symmetric**	**Internal wall** [IW]		Axis, profile	3 diameters, surface area
	**Rim** [R]	different types of rim	Axis, profile, plane	diameter, thickness, height, surface area
	**External wall** [EW]		Axis, profile	3 diameters, surface area
	**Base** [B]	different types of base	Axis, profile, plane	diameter, thickness, height, surface area
	**Wall** ([IW] ∪ [EW])		Axis, profile	thicknesses
**Non-Axially Symmetric**	**Morphological** [M]	different types: hand, lip, bas-relief/stamped decoration, positive decoration, graffiti, foot		
	**Chips** [CH]			surface area
	**Fracture surfaces** [FS]			surface area
	**Constant radius sweep** [CRS]	Axially symmetric Non-axially symmetric	sweep line	radius
